# Reliability and validity of Handwriting Test for Preschool Children (HT-PRE): A new tool to assess the handwriting ability of preschool children aged 5–6 years old in Mainland China

**DOI:** 10.1371/journal.pone.0229786

**Published:** 2020-03-02

**Authors:** Qin Hong, Bei Jiang, Qu Xu, Lei Zhang, Jiaxin Ou, Qingyu Zhang, Nan Li, Jing Wang, Yachun Xie, Jing Hua, Xirong Guo, Meiling Tong, Xia Chi

**Affiliations:** 1 Department of Child Health Care, Women’s Hospital of Nanjing Medical University, Nanjing Maternity and Child Health Care Hospital, Nanjing, Jiang Su, China; 2 Department of Child Health Care, Xuzhou Children’s Hospital, Xuzhou, Jiang Su, China; 3 School of Pediatrics, Nanjing Medical University, Nanjing, China; University of Padova, ITALY

## Abstract

**Background:**

Handwriting ability is related to many neuronal functions, such as visual-perceptual skills, orthographic coding, motor planning and execution, kinesthetic feedback and visual-motor coordination. To date, there is no specific assessment tool for to assess preschool children’s handwriting ability in Mainland China. Our study aimed to develop a tool to assess the handwriting ability of children aged 5–6 years old in Mainland China and to analyze its reliability and validity.

**Methods:**

The investigation comprised three phases: 1) original tool generation, 2) tool revision, 3) reliability analysis (i.e., interrater, test-retest) and validity analysis (i.e., content, criterion).

**Results:**

The sample included a total of 482 children. The internal consistency (Cronbach alpha) was 0.74. The test-retest correlation coefficients ranged from 0.38 to 0.80. As expected, our data showed an improving trend in handwriting, and differences in respect to age and gender. When compared with the ‘handwriting difficulty’ group, each subtest score of children in the ‘normal’ group showed significant differences (*p* < 0.05). The correlation validity, compared with the visual-motor integration development test (VMI), was 0.17–0.52.

**Conclusion:**

The Handwriting Test for Preschool Children (HT-PRE), which is a newly developed handwriting screening tool for preschool children aged 5–6 years old in Mainland China, has displayed a very good internal consistency, acceptable test-retest reproducibility, and good criterion-based validity, and has also shown good application prospects for handwriting difficulty screening in a clinical setting.

## Introduction

The progression of handwriting skills is an essential ingredient for school success [1–3]. Children spend 31%–60% of their school day writing by hand and performing other fine motor tasks [4]. Difficulties in this area can adversely affect academic achievement, and the consequences for academic performance have been well documented [5, 6]. Form errors, which are defined as additions, deletions, or misalignments, in kindergarten children were a predictor of later academic abilities in reading and handwriting [7]. It is clear that handwriting difficulties can overshadow a child’s capabilities in other areas, making success at school less easily attainable. Children with handwriting problems typically find it difficult to keep up with the volume of written work required during the elementary school years. Moreover, insufficient developmental progression in handwriting skills not only leads to the child learning difficulties but also increases the likelihood that the child will develop individual emotional behavioral problems, which can even affect social adaptation and development in adulthood and across the lifespan [2, 8].

Handwriting is a complex perceptual-motor skill that involves a combination of visual-motor coordination abilities, motor planning, cognitive, and perceptual skills, as well as tactile and kinesthetic sensitivities [4, 9–11]. Before children develop mature handwriting skills, they must experience several typical stages, including “drawing”, “invented letters”, “random letters”, “transcription words”, and “developmental spelling” [12]. Due to underdeveloped visual perception abilities, children will exhibit problems with handwriting, such as a slow writing speed, poor accuracy (e.g., multi-stroke, missing strokes, mirror inversion, upside down, etc.), and unevenness between characters or the internal structure [1, 13]. As a result of insufficient motor abilities, children will experience problems such as demonstrating a high level of variability in terms of characters, and they may use different stroke weights [14–16]. In the case of cognitive deficiency, problems such as adding/kneading/replacing letters, parts, or strokes may occur when spelling [8]. Therefore, we can also infer the development of other nerves and muscles by observing handwriting ability.

Under the current education system in mainland China, children in the first grade of primary school are required to write formally not only in school but also after school. In kindergarten, they are required to do some pencil-holding activities, such as painting, scrawling and simple writing, in order to prepare for the written assignments as soon as possible after entering school. As we known, early intervention for the handwriting difficulty is critical at this age, so it is very necessary to understand the development of preschool children's handwriting ability, in order to provide evidence and basic assessment for early intervention. To date, there is no specific assessment tool for to assess preschool children’s handwriting ability in Mainland China. Therefore, it’s meaningful to develop an assessment tool for clinical use.

Most recent research on writing difficulties were carried out in countries that use a phonogram-based language, such as the United States or the Netherlands [4, 17–19]. For example, The Test of Handwriting Skills (THS), developed by Gardner et al. from the United States, was widely used in English-speaking countries[20]. The THS is a comprehensive writing test for children aged 5 years and 0 months to 11 years and 11 months. The children's writing speed, accuracy, and clarity are evaluated by spontaneous writing, dictation, and transcription modes. The test consists in ten subtests, and includes writing all of the English letters in order, dictating and transcribing the selected letters, numbers, words, and so on. THS is based on a rule-based scoring system, such that each individual letter or word can be used as an analytic item, and scores range from 0–3. Thus, it can provide standard scores, scale scores, and percentiles. These results can produce an objective and comprehensive assessment of handwriting skills.

However, each written language has its own unique characteristics and format depending on its origin and development. Alphabetic languages emphasize smoothness and continuity in their written forms [4, 21], whereas characters of the Chinese language contain sharp turns of stroke and demand frequent pen lifts [22]. The problem of handwriting would appear to be more critical, as Chinese characters are typically characterized by their logographic nature and complex multiple stroke sequences and directions. As highlighted by Tan, Hoosain, and Soik, the configurable properties of stroke patterns in Chinese characters do not offer any clue as to where to begin and which stroke to follow when writing a character [23]. Writing in Chinese involves complex geometric figuration and stroke arrangements within a squared-area [24, 25]. Proficient writing of Chinese characters is more difficult than English, as the Chinese language places greater demands on the ability to visually discriminate between fine differences in the form and position of strokes, as well as the ability for spatial organization in order to ensure that characters are written legibly, with the appropriate positioning of strokes and proportioning of radicals [26, 27]. Different compositions, proportions, and orientations of each parts of Chinese characters can lead to the formation of different characters which can produce totally different meanings and pronunciations [28]. Therefore, the method for evaluating the writing ability of phonetic characters is not applicable in the case China though can provide a referential framework that can be used to design our own evaluation tool.

While the literature reveals an extensive body of research that has examined English handwriting, similar studies on Chinese handwriting are relatively limited [1, 26, 29–31]. The Tseng’s Handwriting Speed Test is the most commonly used evaluation tool to assess primary school students’ handwriting speed [32]. It was developed in Taipei to assess the Chinese handwriting speed of students from grade 2 to grade 6. Students were requested to copy a text comprising 475 characters onto an A4-sized sheet of paper with pre-printed grids. Students were given five minutes to complete the task as quickly and as legibly as possible. The number of characters copied was counted manually and the writing speed was calculated. The Handwriting Assessment Checklist was a locally developed and validated handwriting evaluation checklist [33]. It consisted in ten questions that addressed three domains of handwriting, i.e., writing process, writing product, physical and emotional well-being. A newly computerized handwriting evaluation system, the Chinese Handwriting Assessment Tool, was developed by Sutie S.T. Lam in Hong Kong in 2011. Handwriting performance was measured using two constructs, namely, the process of handwriting and the evaluation of the handwriting accuracy [27]. However, all of these currently available Chinese handwriting evaluation tools were designed to assess the handwriting skills of primary school students and were specifically validated for the population and school curriculum of Taipei or Hong Kong, which may not be valid for use in Mainland China. Hong Kong is bilingual, and the spoken languages include Chinese and English, while the written language is traditional Chinese characters. Writing training is undertaken from early childhood, which is much earlier than on the Mainland. Although Mandarin Chinese is the spoken language, the written language in Taiwan is still traditional Chinese characters. Therefore, all these tests are not entirely applicable to children from the Mainland who learns simplified Chinese characters.

Therefore, we planned to design a quick and easy screening tool, called the Handwriting Tools for Preschool Children (HT-PRE), to assess the handwriting ability of preschool children. This tool was utilized with participants who were children aged 5–6 years old, which is the most common age at which to begin writing in Mainland China. According to the analysis of the characteristics of children's writing behavior at this stage, we hope to determine the normative data related to the handwriting ability of children in Nanjing for clinical purposes.

## Materials and methods

Approval to conduct this investigation was granted by the Institutional Review Board at the Nanjing Medical University, Nanjing, China. The study consisted of three separate phases.

### Phase 1: Original tool generation

A multidisciplinary group of handwriting experts was assembled to develop the original measurement tool. The group included neural developmental pediatricians, speech and language pharmacologists, physical therapists, occupational therapists, speech and language therapists, and Chinese language teachers and parents. Based on the basic structure of the English version of the handwriting assessment tool (i.e., "Test of Handwriting Skills", edited by Morrison F. Gardner), and by combining this tool with the main contents which were conducted during school time at the pre-school period, four subtests contained six available scores that constituted the original HT-PRE.

Three sub-components including digit numbers, alphabet letters and simplified Chinese characters were contained in the test. We considered that the assessment of preschool children's writing ability is not only the ability to write Chinese characters, but also the ability to grasp pencils and use fine motor skills to write. Considering that this age group has been exposed to numbers and letters, which are relatively simple and meet the acceptance level of this age, we kept them as important compliments. Simplified Chinese assessment was initially designed to differentiate it from traditional Chinese handwriting assessment, indicating the independence of the test. Both "Spontaneous writing" and "Dictation" are more difficult than “Transcription". They include not only the ability of writing, but also the ability of decoding and working memory. “Names” are almost the first Chinese characters which would be taught at a very young age. They can usually be completed at the age of 5–6, which can be used as a reference for clinical observation. However, individual differences are relatively large; these observations can only be used as a reference, and cannot be included in scoring. The spontaneous writing or dictation of other Chinese characters is too difficult for this age group, so neither of them is included in "Spontaneous writing" and "Dictation" subtests.

**Fig 1:**
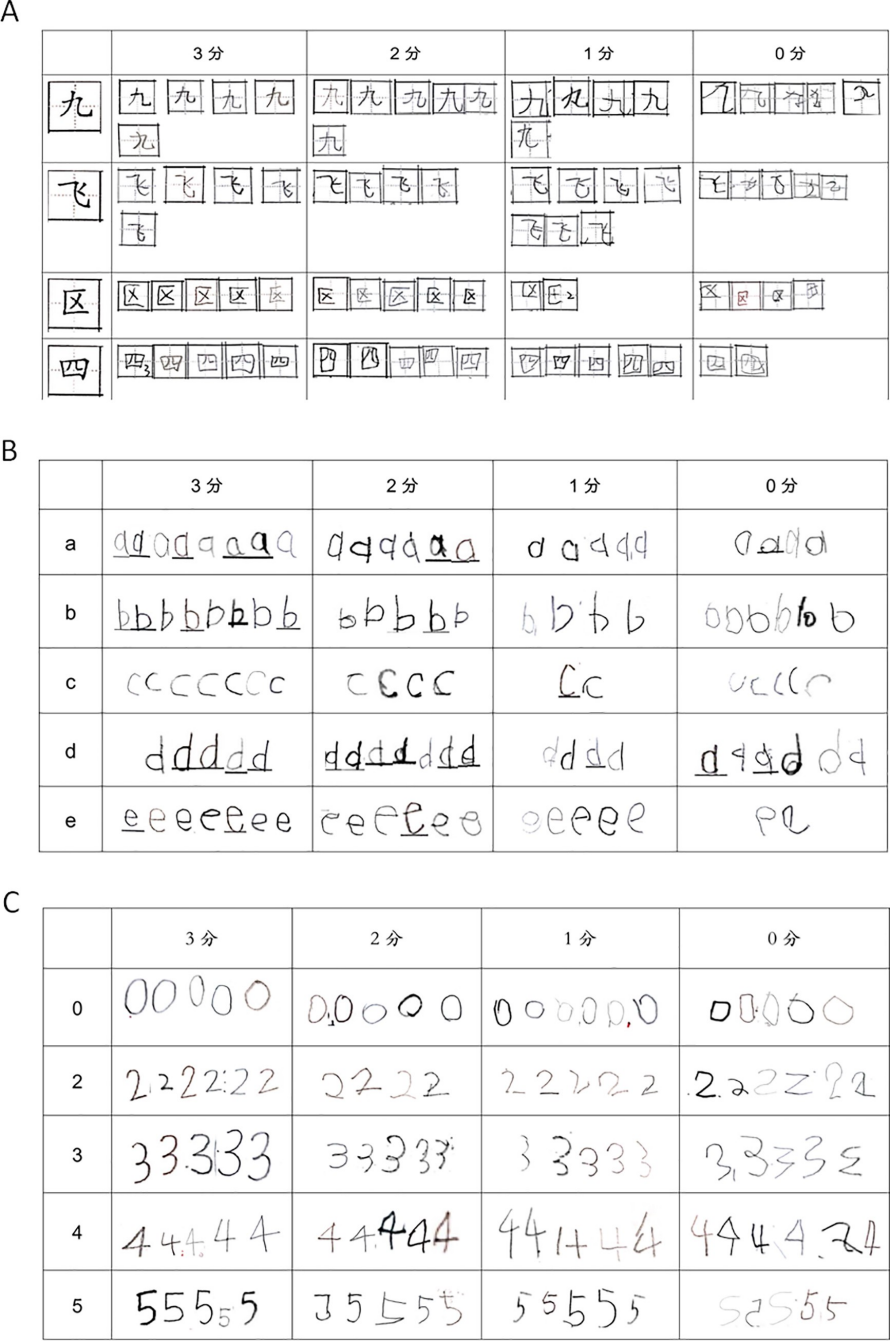
Samples of some parts of scoring criteria. A: Chinese Characters scoring criteria; B: Letters scoring criteria; C: Numbers scoring criteria.

We evaluate handwriting skills by speed, accuracy and construction of written digit numbers, alphabet letters and simplified Chinese characters. Handwriting speed is calculated by the completion time. Handwriting quality contained the accuracy and construction, and was scored manually according to standard scoring criteria. The score for each item is from 0–3, 0 is the lowest score, and 3 is the best. For example, the simplified Chinese characters were scored according to three aspects, numbers and accuracy of strokes, construction of characters and the position in Tin word format. Details were stated in the test’s instructions. The details of the tools are shown in Table 1. Samples of some parts of scoring criteria are shown in Fig 1. Protocols can be seen in dx.doi.org/10.17504/protocols.io.5fmg3k6.

**Table 1:** Description of the Handwriting Test for Preschool Children (HT-PRE) subtests.

**Subtests**	Description	Score	Definition	Rules for score	Raw full marks in each subtest
**Spontaneous Writing**	Write the most familiar contents, including name, age, class, Arabic number (1–9) in sequence by memory.	Ratio Score	the Ratio of the quantity of numbers to completion time (within 20 seconds)	If all 9 digits are correct in 20 seconds that can be marked full marks.	9 points
**Dictation**	Write 10 two-digit numbers the numbers, which is randomly selected in the random number table by listening.	Numbers Score	Total score for writing quality of 10 two-digit number	Each two-digit number is divided into 2 scoring items. The score for each item is from 0–3, 0 is the lowest score, and 3 is the best.	60 points
**Transcription Letters**	24 alphabet letters (except O and I which are easily to be confused with the numbers), which are randomly arranged into two lines, are required to be copied sequentially.	Letters Score	Total score for writing quality of 24 letters	The score for each letter is from 0–3, 0 is the lowest score, and 3 is the best.	72 points
		Letters Time	Writing time of the transcription letters		
**Transcription Chinese Characters**	12 Chinese characters contained all the basic strokes and structures were selected from the 1st to the 4th volume of the primary school textbooks, and were requested to be copied in the same blank standard character grid.	Characters Score	Total score for writing quality of 12 Chinese characters	The score for each character is from 0–3, 0 is the lowest score, and 3 is the best.	36 points
		Characters Time	Writing time of the transcription Chinese characters		

### Phase 2: Original tool revision

A total of 120 children aged 5–6 years old were randomly selected from two kindergarten schools in Nanjing, and 30 children of the same age who had writing difficulties were selected from the children's Psychological Behavior Clinic in Nanjing Maternal and Child Health Care Hospital and were administered the original tool developed for this study. Four expert panel meetings were held to ensure that the revised-version of HT-PRE were valid. At each meeting, panel members received information relating to the progress on the selection of measurements items, the compilation of writing assignments, and the development of the assessment protocol. Comments and suggestions from members were collected individually. Revisions were made based on the suggestions. Having carried out an analysis of difficulty and discrimination, some items were revised again, and the tasks that were difficult to understand or manipulate were deleted or revised.

### Phase 3: Reliability & validity analysis

#### Participants

Using the multi-stage stratified random sampling method, three urban districts (i.e., Qixia District, Qinhuai District, Gulou District), which came from low, media, high income districts in the main urban area of ​​Nanjing respectively, were randomly selected, and two kindergartens were randomly selected in each urban area to recruit children aged 5–6 years old as participants. Prior to the study, all parents or guardians who had participated in the study provided their written informed consent and completed the questionnaire to answer basic information about the children. After obtaining a list of all children aged 5–6 years old, a stratified random sampling was performed according to gender and age requirements. Groups were determined in accordance with each successive six-month age increase. Therefore, the total number of children aged 5–6 years old were divided into four age groups: [5.0, 5.5) age group, [5.5, 6.0) age group, [6.0, 6.5) age group, [6.5, 7.0) age group. The total number of participants was 494, including 242 male (50.21%) and 240 female (49.79%). Exclusion criteria included mental retardation, stunting, low vision, physical disability, or severe trauma. Finally, a total of 482 children were effectively sampled. In addition, 49 cases of children with handwriting difficulties, as recognized by the clinical observation of attending physicians, were randomly selected from the children's psychological behavior clinics at Nanjing Maternal and Child Health Care Hospital in order to participate in this study.

#### Situations, tools and procedure

A comfortable environment with adequate light and good ventilation was requested in order to ensure that the children were attentive to the tasks during the evaluation. While taking the test, children were separated by a sufficient physical distance to avoid copying. Moreover, the examiner confirmed that there were no language symbols in the classroom, such as alphabet posters. Tables and chairs of a suitable size were prepared. When the child was seated on the chair, their feet could be placed flat on the ground.

Tools included the test form, an HB pencil, stopwatch, and no eraser. To avoid any non-essential linguistic symbols, which may affect children's handwriting, no written instructions or requirements were specified in the test manual, and only non-verbal symbols (e.g., cartoon pictures) could be used to mark the page and number items.

Before the test, parents completed the "Children's Basic Situation Questionnaire" so that necessary information about the participants could be gathered, including the child's age, gender, medical history, and trauma history. Three tests were conducted an in sequence outlined in the "Combined Raven Test", “HT-PRE”, and “Developmental Test of Visual-Motor Integration (VMI)”. The Combined Raven Test, which is a non-literal intelligence test, was first conducted one-on-one in the classroom. The participant was requested to select the most suitable image. The original score was calculated, and then transferred into IQ test. Children with an IQ < 70 were excluded from the study. The HT-PRE was then conducted one-on-one. The VMI test was carried out in the classroom in groups comprising five children. The VMI is a screening tool that was designed by K. E. Beery for the early identification and prediction of children's learning and behavioral problems. It can be used with children who are above two years old. The main purpose of the VMI is to evaluate the ability for visual-motor integration by referring to children's geometrical figures, reflecting the eye-hand coordination status. This test has been confirmed to have a stable reliability, and can be used as a standardized reference to evaluate children's handwriting ability. Finally, “The Individual Record Form” was used to record all special behavior observed during testing.

#### Reliability analysis

Internal consistency was assessed using Cronbach’s correlation coefficient. The intra-class correlation coefficient (ICC) was calculated to evaluate the test-retest reliability. Two weeks later, 27 children from different age groups and different kindergartens were randomly selected for test-retest reliability under the same situation. Such a test-retest interval was long enough to prevent fatigue, memory, or learning effects, and short enough to avoid genuine changes in the measured variables[34].

#### Validity analysis

Construct validity refers to the reasonable validity of the internal structure of the scale. The construct validity of the HT-PRE is mainly tested by comparing the scores between different age groups and genders. Differences in the HT-PRE test between the ‘difficulty’ groups and the ‘normal’ groups were compared using an independent t-test. Therefore, we believed the discriminatory ability of our tool for children with handwriting difficulties was acceptable.

Criterion-related validity was reflected in the relationship between the research tool and other measurement standards. The higher the correlation coefficient is, the more objective and fairer the evaluation results. In this study, Pearson’s correlation coefficients were used to measure the correlation between the VMI and HT-PRE subtest scores for criterion-related validity.

#### Data analysis

All data were collected, coded and recorded using SPSS Statistics Version 23.0. The quantitative data (or measurement data) that were described in terms of the mean ± standard deviation or median were compared using an independent t-test. The qualitative data (or count data) were described in percentage terms and were compared using a chi-squared test. All tests were two-sided, and *p* < 0.05 was considered statistically significant.

## Results

### Original tool development

A total of 120 randomly selected children and 30 children with writing difficulties participated in our predictive research, the demographic data were shown in Table 2 and Table 3 respectively. After being assessed, all participants were classified, and children who scored in the top 27% were considered as the ‘higher score’ group and those whose scores were in the bottom 27% were assigned to the ‘lower score’ group. The distinction means the difference between these two groups, calculated using the formula D = (XH-XL)/W (XH refers to average score of the ‘higher score’ group, XL refers to average score of the ‘lower score’ group, W refers to total score). The difficulty coefficient was calculated according to the formula P = x. /xmax × 100% (x. refers to the average score, xmax refers to the highest score). As shown in Table 4, the distinction ranged from 0.46–0.60 and the difficulty ranged from 0.50 to 0.74, which was suitable for the screening test.

**Table 2:** The demographic data of the normal group.

group	5.0 y~ (n = 30)	5.5y~ (n = 30)	6.0y~ (n = 30)	6.5y~ (n = 30)	Total (n = 120)
**male**	15(12.50%)	15(12.50%)	15(12.50%)	15(12.50%)	60(50.00%)
**female**	15(12.50%)	15(12.50%)	15(12.50%)	15(12.50%)	60(50.00%)

**Table 3:** The demographic data of the handwriting difficulty group.

group	5.0 y~ (n = 9)	5.5y~ (n = 7)	6.0y~ (n = 7)	6.5y~ (n = 7)	Total (n = 30)
**male**	4(13.33%)	4(13.33%)	3(10.00%)	3(10.00%)	14(46.67%)
**female**	5(16.67%)	3(10.00%)	4(13.33%)	4(13.33%)	16(53.33%)

**Table 4:** Difficulty and distinction of original tool.

	Ratio Score	Numbers Score	Letters Score	Characters Score
**Difficulty**	0.73	0.74	0.71	0.50
**Distinction**	0.60	0.53	0.53	0.46

### Reliability analysis

A total of 482 participants (i.e., 242 males and 240 females) between 5 and 6 years old from six kindergartens participated in our formal study and constituted a ‘normal’ group. Each group from a kindergarten contributed to 16.67% of the total number of participants. All participants were distributed among four age groups, see Table 5.

**Table 5:** The demographic data of the subjects in norm.

Group	5.0 y~ (n = 132)	5.5y~ (n = 124)	6.0y~ (n = 125)	6.5y~ (n = 101)	Total (n = 482)
**Male**	67(13.90%)	63(13.07%)	61(12.66%)	51(10.58%)	242(50.21%)
**Female**	65(13.49%)	61(12.66%)	64(13.28%)	50(10.37%)	240(49.79%)

The internal consistency of the HT-PRE indicated a high reliability (Cronbach alpha = 0.74) [34] [35].

As shown in Table 6, the ICC of the test-retest reliability coefficient ranged from 0.38 to 0.80. The letter scores showed a value of 0.80, indicating a high reliability; Chinese character time and character scores had values of 0.62 and 0.66, respectively, indicating a moderate reliability. The numbers score and letters times had values of 0.52 and 0.45, respectively, which indicated a moderate positive correlation. The test-retest reliability of the ratio score was 0.38, indicating poor reliability.

**Table 6:** Intra-class correlation coefficients (ICC) of each subtest in the test-retest.

	Test($\bar{x}$±SD)	Retest($\bar{x}$±SD)	r	*p*
**Ratio Score**	6.12±1.73	6.85±2.50	0.38	0.06
**Numbers Score**	46.46±10.82	47.89±11.23	0.52	<0.05
**Letters Score**	49.88±18.13	53.93±16.98	0.80	<0.01
**Character Score**	18.19±6.85	17.33±8.03	0.66	<0.01
**Letters Time(s)**	203.04±75.48	298.15±188.76	0.45	<0.01
**Chinese Character Time(s)**	258.61±149.50	279.56±103.94	0.62	<0.01

### Validity analysis

The values of the handwriting quality (score) and handwriting speed (time) of each subtest were expressed as the mean ± SD, see Table 7, Table 8.

**Table 7:** Comparison of scores in different age groups.

Age group	5.0y~	5.5y~	6.0y~	6.5y~	F	P
**Ratio score**	7.52±2.10	11.36±1.94	11.94±2.44	12.50±3.85	37.38	<0.01
**Numbers score**	42.38±5.43	45.02±4.26	46.19±5.78	47.05±4.94	80.13	<0.01
**Letters score**	46.49±6.31	50.44±6.68	53.05±4.51	56.24±4.23	112.56	<0.01
**Character score**	16.66±4.87	18.09±3.00	19.00±3.80	19.68±4.93	37.38	<0.01
**Letters time(s)**	235.05±115.29	185.44±103.08	157.70±100.90	146.42±92.79	21.28	<0.01
**Chinese character time(s)**	353.19±201.82	353.57±135.43	293.82±118.41	264.84±92.96	10.68	<0.01

**Table 8:** Comparison of scores in different gender and different age groups.

Age group	Items	Male	Female	t	P
**5.0y~**	Ratio score	4.55±2.75	5.00±2.25	-1.03	0.30
	Numbers score	29.95±18.62	32.98±17.83	-0.95	0.35
	Letters score	35.64±18.01	35.88±16.93	-0.08	0.94
	Character score	11.61±7.07	12.67±7.06	-0.86	0.39
	Letters time(s)	249.23±126.37	220.25±100.52	1.44	0.15
	Chinese character time(s)	321.71±201.94	379.97±196.55	-1.66	0.09
**5.5y~**	Ratio score	6.70±2.18	7.27±1.69	-1.64	0.10
	Numbers score	44.47±13.31	49.77±9.71	-2.56	<0.05
	Letters score	49.13±16.40	55.73±9.65	-2.76	<0.05
	Character score	16.59±6.28	19.82±4.97	-3.20	<0.05
	Letters time(s)	212.05±136.00	171.21±72.78	2.11	<0.05
	Chinese character time(s)	387.86±148.29	320.94±116.75	2.80	<0.05
**6.0y~**	Ratio score	7.30±1.70	7.34±1.67	-0.14	0.89
	Numbers score	48.38±10.04	51.79±5.79	-2.29	<0.05
	Letters score	55.20±13.91	60.41±7.23	-2.58	<0.05
	Character score	19.00±5.72	21.90±3.68	-3.31	<0.01
	Letters time(s)	161.75±62.86	150.36±71.00	0.93	0.35
	Chinese character time(s)	295.32±100.86	289.73±132.99	0.26	0.80
**6.5y~**	Ratio score	7.54±1.74	7.60±1.89	-0.16	0.87
	Numbers score	50.25±8.15	51.88±8.97	-0.97	0.33
	Letters score	60.92±8.90	62.29±7.46	-0.85	0.40
	Character score	21.10±5.21	22.65±5.20	-1.53	0.13
	Letters time(s)	151.08±68.52	146.02±57.68	0.41	0.69
	Chinese character time(s)	268.40±104.08	269.21±82.39	-0.04	0.97

**Fig 2:**
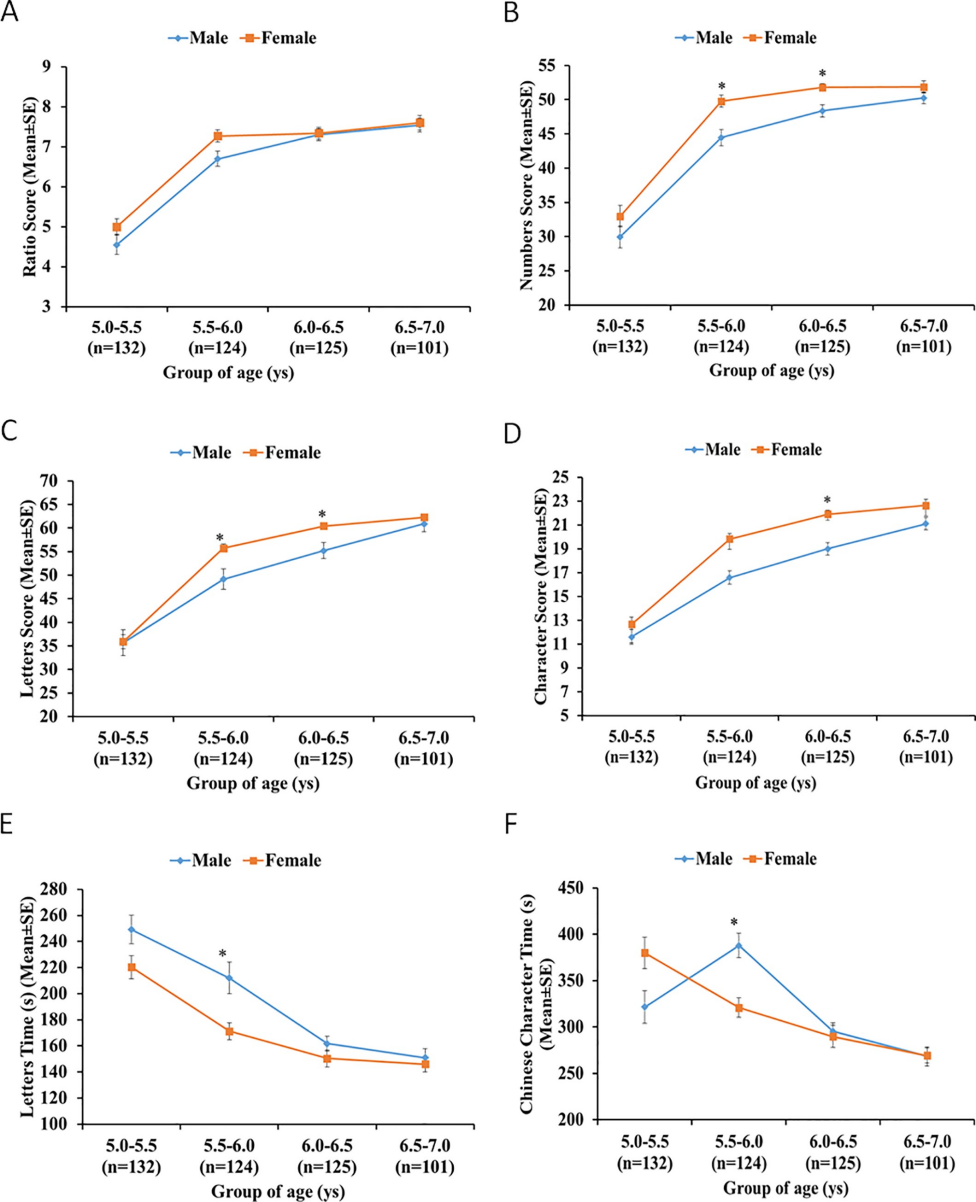
Scores of each subtest by different age groups and gender. A: Ratio Score by different age groups and gender; B: Numbers Score by different age groups and gender; C: Letters Score by different age groups and gender; D: Characters Score by different age groups and gender; E: Letters Time by different age groups and gender; F: Chinese Characters Time by different age groups and gender.

As expected, Fig 2 shows that for both male and female groups, the handwriting score of each subtest increased, while the handwriting time decreased as children aged, which indicates an improvement in handwriting skills. The gender analysis of handwriting ability showed that there was no significant difference in the writing score and writing time in the youngest group [5.0–5.5) and the oldest group [6.5–7.0). All scores related to the qualities of writing revealed differences between males and females in both the [5.5–6.0) and [6.0–6.5) age groups.

**Fig 3:**
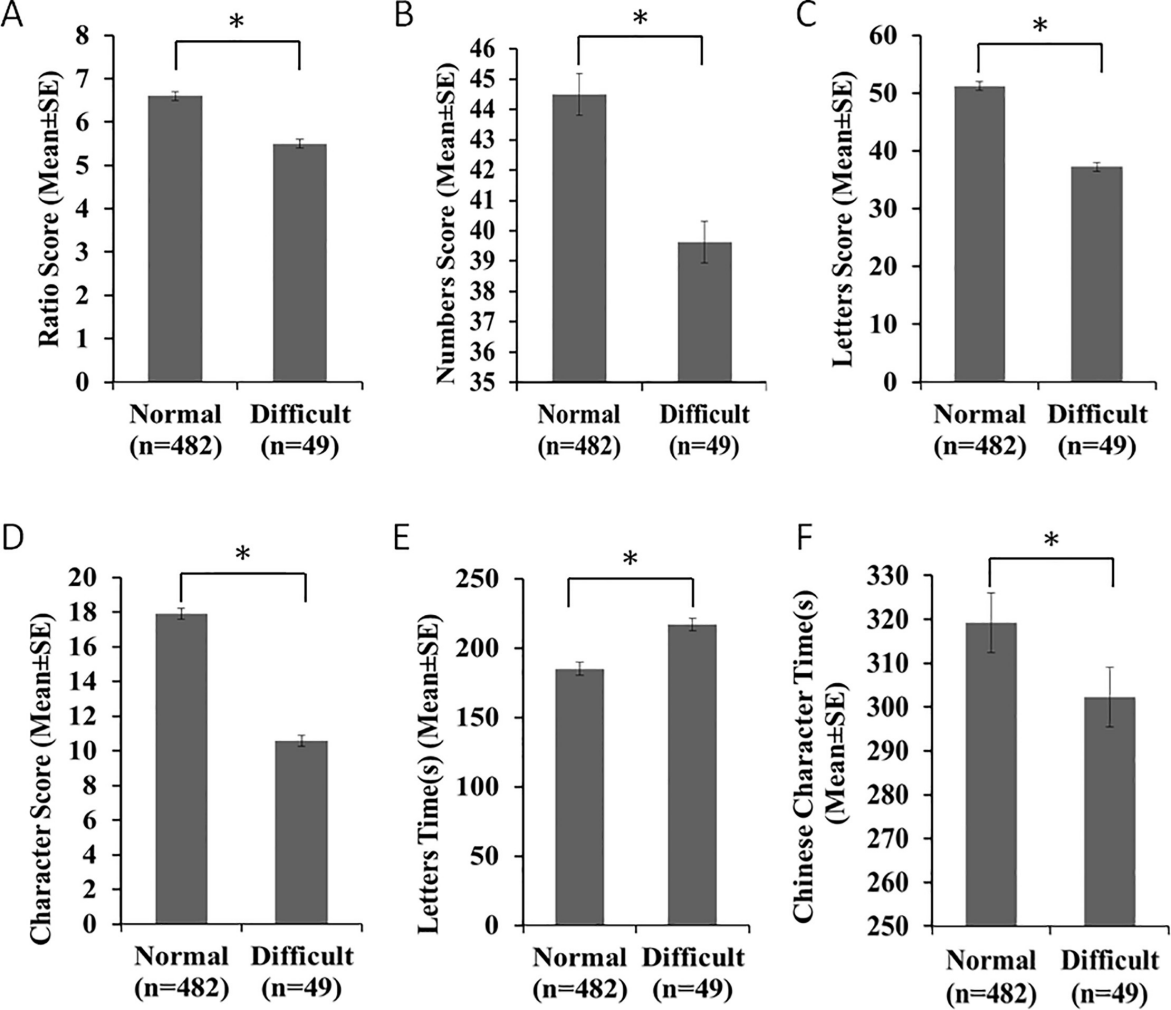
Score differences between normal group and handwriting difficult group. A: Ratio Score Difference between normal group and handwriting difficult group; B: Numbers Score Difference between normal group and handwriting difficult group; C: Letters Score Difference between normal group and handwriting difficult group; D: Characters Score Difference between normal group and handwriting difficult group; E: Letters Time Difference between normal group and handwriting difficult group; F: Chinese Characters Time Difference between normal group and handwriting difficult group.

The analysis also showed significant differences between the ‘normal’ group (**n = 482**) and the ‘handwriting difficulty’ group (**n = 49**) using our tool (*p* < 0.05), as shown in Table 9 and Fig 3.

**Table 9:** Scores different between normal group and handwriting difficult group.

	Normal group (mean ± SD, n = 482)	Control group (mean ±SD, n = 49)	t	P
**Ratio score**	6.60±2.32	5.50±2.35	0.38	<0.01
**Numbers score**	44.49±14.98	39.63±11.61	0.52	<0.01
**Letters score**	51.22±16.65	37.24±16.22	0.80	<0.01
**Character score**	17.91±6.97	10.57±7.63	0.66	<0.01
**Letters time(s)**	185.04±99.53	216.73±107.04	0.45	<0.05
**Chinese character time(s)**	319.19±149.57	302.22±113.55	0.62	<0.01

A comparison of the HT-PRE and the VMI shows that criterion-related validity ranged from 0.17 to 0.52, as illustrated in Table 10.

**Table 10:** Pearson’s correlation coefficient matrix of HT-PRE and VMI.

		Ratio Score	Number Score	Letters Score	Chinese Score
VMI	r	0.17	0.38	0.52	0.43
	*P*	<0.01	<0.01	<0.01	<0.01

## Discussion

This tool is to assess the child's handwriting skill, which is a small but indispensable part of learning ability. Some children with developmental coordination disorder have deficiency of handwriting skills in the early stage of development. This difficulty persists after enrolling school, which affects the completion of assignments in and after school. As we know, the current education in China, whether in class or after class, has considerable written assignments. The quality and speed of assignments not only affect students' performance in school, teachers' evaluation and learning, but also students' self-evaluation, even peer evaluation. Therefore, we wonder it has a direct impact on children's school performance, not limited to the impact on academic performance.

Throughout the literature, handwriting performance is often assessed in terms of two dimensions, namely, legibility and speed. Legibility consists of a variety of elements such as errors in letter formation, reversals of letters, spacing between letters and words, letter size, slant, and alignment [36, 37]. In fact, through factor analyses, studies have indeed found that these elements relating to legibility can be grouped separately and classified into a few factors. In the alphabetic system, four factors (i.e., letter formation, spacing, alignment, and size) have been identified [30]. Similarly, in the Chinese handwriting of school-aged children, three factors have been deemed relevant, i.e., construction (e.g., spacing between characters, size of characters), accuracy (e.g., adding or missing strokes), and directionality (e.g., reversal of components) [2, 38]. Writing speed refers to the time required to complete a writing task. Depending on the results of the writing assessment, an accurate intervention could be promptly provided, and could be sufficient to improve academic achievement upon entry to elementary school.

### Rationale of the HT-PRE

At present, the handwriting skills of school-age children in China mainly include three forms: spontaneous writing, dictation and transcription. An individual’s writing ability reflects the comprehensive coordination of motor functioning, visual perception, and cognition. By carrying out a survey of the teachers and parents of kindergarten children, we learned that pre-school children, whether at home or at school, most commonly learn numbers, letters, and their own name. Therefore, our tool involves not only different processes of writing, but is also within the scope of preschool children's abilities.

In the spontaneous writing subtest, most preschool children can only attempt to write some Arabic numerals from 1–9 or their personal names, particularly during their first year of learning to write. Due to the different levels of difficulty associated with children's names, this item is not included in the total score in the following normative study that we conducted, though it can be used as a pre-selected topic to encourage familiarity between the participants and the test administrators.

In the original dictation phase, only digital dictation can be completed, as the other tasks are too difficult. Therefore, according to the actual writing ability of children aged 5–6 years old, both spontaneous writing and dictation content include writing numbers.

The transcription subtest consists of two parts, i.e., transcribing letters and transcribing Chinese characters. The 24 letters subtest is an original test derived from the "Test of Handwriting skills", which is randomly arranged in the form of two lines that should be copied sequentially. This item has been shown to be a reasonable and effective test item. The Chinese characters subtest was self-designed. According to the different forms of the characters, the Chinese characters are divided into two types, i.e., single-character and combined-character. According to the structures of combined-characters, they are divided into four types, i.e., upper and lower, left and right, inside and outside, and character structure. All of these 12 Chinese characters were selected from the first four volumes of Chinese elementary textbooks, which contain all of the basic strokes and structures of Chinese characters. As Chinese characters are organized using different parts and are arranged side-by-side, the structures are complex and the distances between parts are unequal. Therefore, the Chinese character transcription test requires more in terms of the child’s capacity for visual discrimination, fine motor skills, and integrated processing. Our test offered a good method of assessing handwriting in a Chinese environment. Predictive research has also shown that the degree of difficulty and the discrimination of all subtests were acceptable as a screening test.

At the same time, the evaluations provided by the professors of linguistics, the therapists, psychologists in the Children's Physical Development Evaluation Center as well as the teachers and parents of kindergarten children ensured the rationality, feasibility, and comprehensiveness of the HT-PRE. Therefore, the HT-PRE, which is based on the writing development of children aged 5–6 years old in China, could be a tool by which to assess the progression of handwriting ability in Chinese children.

### The validity and reliability of the HT-PRE

Cronbach’s alpha coefficient was used to test for internal consistency, and this test had a high internal consistency. The ICC was calculated to evaluate the test-retest reliability. ICC data ranged from 0 (no reliability) to 1 (perfect reliability). Scores that ranged between 0.9–1.0 were considered to indicate very high reliability, 0.7–0.9 indicated high reliability, 0.5–0.7 indicated moderate reliability, and 0.3–0.5 suggested low reliability[35]. We found that the test-retest reliabilities of transcription letters and Chinese characters showed a strong correlation. Dictation number test-retest reliability indicated a moderate correlation, while the reliability of spontaneous writing was not as good as expected. That may be due to a practice effect in respect to the children's first attempt at writing and learning, which could improve or even be mastered skillfully after two weeks of practice. As an important type of writing task, spontaneous writing is indispensable in a writing ability test. While considering the actual writing ability of children aged 5–6 years old, there is no alternative test. Therefore, the spontaneous number writing subtest should be retained, and the weight of the ratio scores of this subtest in the normative study will be adjusted. The writing time test-retest reliability is 0.45–0.62. The children were requested to again perform the same tasks for 5–6-year-olds after 2 weeks, and to do so without any specific reward or sense of achievement. Therefore, their attention and motivation may decrease during the retest, thus resulting in fluctuations in writing speed.

The construct validity results of the HT-PRE were consistent with the other relevant studies. As expected, the HT-PRE scores of every subtest increased as the children aged, indicating an improvement in handwriting quality. The gender differences in handwriting were reported differently. There were no gender differences in performance on the VMI or on the SCRIPT, while in a handwriting speed norm study contained 1525 children in Taiwan, girls wrote faster than boys in grades 3,4, and 5 and gender differences were also found in English handwriting performance evaluations in kindergarten children. Our studies showed that there was no significant difference in the writing score and writing time in both the youngest group [5.0–5.5) and the oldest group [6.5–7.0). We analyzed the reasons for this finding: In China, when a child is 5–5.5 years old, the child has just entered the middle kindergarten class, which means that the child’s writing ability is still the same in terms of maintaining a correct sitting posture and holding a pencil correctly. Practicing writing mainly involves color painting, which makes it difficult to write complex characters, regardless of the child’s gender. Children in the [6.5–7.0) group are about to enter elementary school. Most parents pay attention to their children's learning-related abilities, and push their children to practice and learn simple letters and Chinese characters. Therefore, no difference was observed between males and females in this group. It can be seen that all of the scores related to the qualities of writing show gender differences in both the [5.5–6.0) and [6.0–6.5) age groups. In accordance with the results of Chinese handwriting skills, females performed better than males not only in terms of the writing quality but also in respect to the speed of handwriting during this phase[32, 39, 40]. The results were also supported by the literature [7, 41], which indicated that boys may need more time to develop fine motor skills [42, 43]or other handwriting-related skills. Therefore, we considered that different methods using in the studies might induced different conclusions. Our method seemed more sensitive to evaluate the gender difference of handwriting performance in 5.5–6.5 age groups. It should be modified in younger and older groups in the future. Furthermore, we found that the qualities of handwriting in normal children were much higher than in children with handwriting difficulties, using our tool. But interestingly, we also found that normal children spent significantly less time in "Letters times" than children with handwriting difficulites, and more time in "character times". We speculate that because Chinese characters are more complex than letters, it may take more time for normal children to write better. Some children with handwriting difficulties spend less time because they might write fewer strokes, or were sloppy doing this.

The VMI test was used to validate the calibration-related validity of the HT-PRE. Till now these is no golden standard for diagnosis of handwriting difficulty, VMI, mainly evaluate the ability of visual-motor integration according to children's geometrical figures, reflecting the eye-hand coordination, is the only tool which can be used at this age and related to handwriting ability. The results showed a medium correlation between VMI and transcribing letters. Numbers and letters in our test composed of simple lines are similar to drawings in VMI, so that the validities of transcribing numbers and letters were now acceptable. However, the results revealed a low correlation between VMI and dictation of numbers and transcription of Chinese characters. VMI mainly evaluates handwriting ability dependent on visual signals, while dictation numbers assess handwriting ability dependent on auditory signals. Therefore, the signal sources were completely different, a low correlation was acceptable. As identically using visual signals, the main difference between the transcription of Chinese characters and VMI lies in the object that is being written. Chinese character handwriting not only involves more strokes, but also requires more spatial structure concepts. Therefore, transcribing Chinese characters requires more proficient handwriting skills involving the visual signal, which explained the low correlation between VMI and transcription Chinese characters.

Furthermore, we found positive but low correlations between HT-PRE and VMI, which indicated that VMI might indeed not be a suitable tool for Chinese handwriting evaluation. Therefore, it is beneficial to develop a writing assessment tool for Simplified Chinese. The results indicated that the letter score and character score are the most reliable and preferred methods to evaluate handwriting skills at this age. As such, in the normative study, we assigned them greater weights when determining the total score.

## Conclusion

The HT-PRE is a self-designed handwriting assessment tool for 5–6 years old preschool children in China. This tool has a very good internal consistency, and has shown acceptable test-retest reproducibility, and good criterion-based validity. We also carried out a normative study to determine the criteria for clinical purposes. Furthermore, the average assessment time of 20 minutes and the portable nature of this tool highlight its clinical focus. The HT-PRE may be utilized as a clinical screening test for handwriting difficulties.

### Limitation

This study sampled 482 children in three urban districts in main urban area of ​​Nanjing, which represented the ability of main urban children in Nanjing, may not represent a larger population in other areas. However, it’s meaningful to clinical purpose, and we hope to expand the samples with the increase of funds input in further study.

HT-PRE is a newly developed handwriting screening tool for preschool children aged 5–6 years old in Mainland China, which may have some shortcomings, and we will keep updating them in the future.

## Supporting information

S1 Table Retest data. Two weeks later, 27 children from different age groups and different kindergartens were randomly selected for test-retest reliability under the same situation.
(PDF)

S2 Table Norm data. A total of 482 participants (i.e., 242 males and 240 females) between 5 and 6 years old from six kindergartens participated in our formal study and constituted a norm.
(PDF)

S3 Table Handwriting difficult data. 49 cases of children with handwriting difficulties, as recognized by the clinical observation of attending physicians, were randomly selected from the children's psychological behavior clinics at Nanjing Maternal and Child Health Care Hospital in order to participate in this study.
(PDF)
