# Supplementary material for: Reliability and validity of Handwriting Test for Preschool Children (HT-PRE): A new tool to assess the handwriting ability of preschool children aged 5–6 years old in Mainland China
Source: PLoS One. 2020 Mar 2;15(3):e0229786. doi: 10.1371/journal.pone.0229786 (PMC7051084; doi:10.1371/journal.pone.0229786)
Supplement: S1 Table — Two weeks later, 27 children from different age groups and different kindergartens were randomly selected for test-retest reliability under the same situation. (PDF) [file pone.0229786.s001.pdf]

Number	number	numbertime	number score	letter time-minute	letter time-second	letter score	characters time-minute	characters time-second	characte score
3020236.00	6.00	20.00	60.00	2.00	46.00	72.00	4.00	32.00	24
3020127.00	6.00	20.00	50.00	2.00	3.00	55.00	2.00	54.00	20
2010235.00	9.00	14.00	47.00	1.00	9.00	64.00	2.00	24.00	26
3020106.00	7.00	20.00	60.00	2.00	22.00	72.00	5.00	10.00	26
3020228.00	8.00	20.00	58.00	33.00	33.00	68.00	5.00	55.00	24
3020207.00	6.00	20.00	52.00	2.00	46.00	69.00	5.00	10.00	22
3020218.00	9.00	19.00	49.00	1.00	59.00	63.00	3.00	42.00	19
3020113.00	5.00	20.00	51.00	4.00	9.00	46.00	6.00	31.00	13
3020120.00	8.00	20.00	45.00	4.00	57.00	20.00	0.00	0.00	0
3020110.00	7.00	20.00	44.00	3.00	4.00	39.00	4.00	25.00	5
3020212.00	7.00	20.00	52.00	2.00	35.00	57.00	3.00	43.00	18
3020136.00	9.00	18.00	43.00	32.00	33.00	66.00	3.00	28.00	20
3020210.00	8.00	20.00	56.00	2.00	28.00	59.00	5.00	9.00	14
1010232.00	9.00	13.00	52.00	3.00	18.00	58.00	4.00	20.00	26
2020110.00	9.00	18.00	46.00	2.00	58.00	40.00	8.00	9.00	11
2020129.00	9.00	19.00	49.00	1.00	49.00	56.00	3.00	50.00	6
2020228.00	7.00	20.00	58.00	2.00	20.00	70.00	5.00	23.00	30
2020225.00	9.00	15.00	51.00	1.00	37.00	61.00	3.00	39.00	27
2020102.00	8.00	20.00	49.00	2.00	8.00	69.00	6.00	5.00	21
2020120.00	9.00	20.00	0.00	2.00	58.00	58.00	4.00	39.00	13
2020207.00	4.00	20.00	41.00	4.00	23.00	57.00	7.00	49.00	18
2020221.00	9.00	14.00	51.00	1.00	14.00	68.00	3.00	27.00	23
2020226.00	7.00	20.00	58.00	2.00	3.00	64.00	3.00	28.00	22
1020206.00	0.00	0.00	46.00	2.00	31.00	41.00	4.00	27.00	17
1020201.00	4.00	20.00	43.00	3.00	5.00	32.00	5.00	25.00	9
1020202.00	6.00	20.00	39.00	3.00	15.00	19.00	4.00	25.00	12
1020104.00	0.00	0.00	43.00	4.00	7.00	13.00	7.00	39.00	2
