# Supplementary material for: Reliability and validity of Handwriting Test for Preschool Children (HT-PRE): A new tool to assess the handwriting ability of preschool children aged 5–6 years old in Mainland China
Source: PLoS One. 2020 Mar 2;15(3):e0229786. doi: 10.1371/journal.pone.0229786 (PMC7051084; doi:10.1371/journal.pone.0229786)
Supplement: S2 Table — A total of 482 participants (i.e., 242 males and 240 females) between 5 and 6 years old from six kindergartens participated in our formal study and constituted a norm. (PDF) [file pone.0229786.s002.pdf]

Number	sex	number time	number score	letter time-second	characters time-minute	characters score	VMI
1010245	2.00	20.00	60	18.00	4.00	32	26.0
2010146	1.00	13.00	52	18.00	4.00	28	25.0
1020125	1.00	15.00	56	13.00	2.00	24	25.0
1020118	1.00	20.00	50	48.00	6.00	19	24.0
2010242	2.00	20.00	59	10.00	4.00	23	22.0
3010129	1.00	20.00	39	19.00	5.00	20	22.0
2010129	1.00	20.00	56	0.00	5.00	18	23.0
3010218	2.00	16.00	56	56.00	3.00	22	22.0
3010245	2.00	20.00	52	41.00	3.00	20	22.0
3010208	2.00	14.00	49	0.00	4.00	19	23.0
3010243	2.00	20.00	57	48.00	6.00	28	24.0
1020136	1.00	20.00	51	28.00	4.00	29	23.0
3020129	1.00	20.00	59	59.00	4.00	24	21.0
3020135	1.00	17.00	59	31.00	1.00	24	21.0
3010128	1.00	20.00	44	14.00	3.00	16	22.0
1020232	2.00	20.00	57	36.00	3.00	26	22.0
3010132	1.00	20.00	57	49.00	3.00	26	23.0

3010241	2.00	20.00	49	21.00	2.00	29	22.0
3020106	1.00	20.00	57	50.00	6.00	29	23.0
2020232	2.00	20.00	52	50.00	5.00	29	25.0
2020138	1.00	12.00	51	45.00	1.00	26	24.0
2020140	1.00	20.00	56	34.00	4.00	31	23.0
1010143	1.00	13.00	53	44.00	3.00	33	23.0
2020127	1.00	20.00	52	37.00	7.00	23	23.0
3010237	2.00	20.00	59	50.00	4.00	27	22.0
1020230	2.00	18.00	57	49.00	2.00	20	21.0
1020124	1.00	18.00	56	46.00	2.00	22	21.0
1010241	2.00	16.00	49	40.00	3.00	21	21.0
2010241	2.00	20.00	59	10.00	5.00	22	20.0
3020201	2.00	20.00	60	23.00	3.00	25	19.0
3020211	2.00	20.00	58	28.00	4.00	24	22.0
2010144	1.00	20.00	57	17.00	6.00	21	20.0
3010232	2.00	20.00	54	57.00	4.00	17	20.0
1020135	1.00	20.00	51	34.00	3.00	18	22.0
3010135	1.00	20.00	59	11.00	5.00	23	23.0
2010245	2.00	15.00	53	31.00	2.00	19	21.0
3020240	2.00	20.00	54	14.00	4.00	25	25.0

2020228	2.00	20.00	60	50.00	8.00	26	24.0
3010219	2.00	20.00	57	20.00	4.00	20	24.0
2020211	2.00	20.00	51	42.00	4.00	24	22.0
3010244	2.00	20.00	58	7.00	4.00	25	21.0
2010136	1.00	20.00	55	42.00	8.00	20	20.0
1020211	2.00	20.00	60	53.00	7.00	24	19.0
3020224	2.00	20.00	58	15.00	9.00	24	19.0
3010115	1.00	20.00	57	19.00	7.00	14	19.0
1020137	1.00	15.00	52	13.00	2.00	21	19.0
1020119	1.00	20.00	48	20.00	5.00	26	19.0
1020227	2.00	18.00	47	21.00	2.00	26	19.0
2010121	1.00	20.00	49	54.00	2.00	22	18.0
3020105	1.00	20.00	58	54.00	4.00	25	17.0
1010107	1.00	20.00	57	7.00	5.00	15	17.0
3020235	2.00	20.00	54	33.00	6.00	25	18.0
2020215	2.00	20.00	51	12.00	5.00	24	18.0
1010243	2.00	20.00	60	40.00	3.00	32	18.0
2020221	2.00	20.00	51	20.00	4.00	18	18.0
2010122	2.00	15.00	57	33.00	3.00	24	20.0
1020216	2.00	20.00	53	48.00	3.00	20	20.0

2020227	2.00	20.00	58	51.00	6.00	26	21.0
2010128	1.00	20.00	56	34.00	10.00	20	21.0
2010237	2.00	12.00	54	58.00	3.00	27	21.0
1020224	2.00	20.00	55	25.00	7.00	20	19.0
1020128	1.00	20.00	52	6.00	2.00	29	19.0
2010139	1.00	20.00	52	54.00	0.00	18	18.0
2020123	1.00	20.00	50	46.00	5.00	15	18.0
3010226	2.00	19.00	49	39.00	3.00	18	18.0
3010108	1.00	20.00	58	52.00	4.00	19	19.0
2010243	2.00	16.00	53	59.00	3.00	24	19.0
2020233	2.00	20.00	50	25.00	4.00	25	19.0
3020226	2.00	0.00	0	33.00	5.00	25	19.0
1020226	2.00	20.00	49	42.00	6.00	20	16.0
1010213	2.00	20.00	59	51.00	4.00	32	20.0
3020127	1.00	20.00	51	12.00	6.00	28	21.0
3020206	2.00	20.00	59	14.00	7.00	24	20.0
3020228	2.00	20.00	58	32.00	7.00	22	20.0
3020133	1.00	20.00	51	3.00	3.00	28	20.0
3020131	1.00	15.00	49	27.00	6.00	16	20.0
3020231	2.00	18.00	52	50.00	3.00	24	19.0

2020225	2.00	20.00	51	4.00	5.00	24	22.0
3010209	2.00	13.00	48	57.00	2.00	19	22.0
1020215	2.00	19.00	49	32.00	3.00	17	24.0
1020222	2.00	20.00	52	46.00	4.00	20	19.0
3020214	2.00	20.00	18	6.00	6.00	25	20.0
2010145	1.00	20.00	52	4.00	3.00	15	20.0
2010244	2.00	19.00	45	32.00	3.00	19	19.0
2010143	1.00	15.00	48	34.00	3.00	22	17.0
3020225	2.00	20.00	45	49.00	7.00	23	20.0
1010204	2.00	20.00	54	0.00	0.00	23	20.0
3010220	2.00	14.00	51	35.00	3.00	21	20.0
1020123	1.00	17.00	55	37.00	4.00	17	21.0
3010223	2.00	19.00	57	47.00	6.00	18	20.0
1020130	1.00	15.00	49	39.00	3.00	21	20.0
2010221	2.00	20.00	47	56.00	5.00	22	20.0
3010240	2.00	20.00	40	38.00	7.00	20	18.0
3010217	2.00	20.00	57	1.00	5.00	18	17.0
2010134	1.00	20.00	56	40.00	6.00	16	17.0
2020216	2.00	20.00	56	45.00	5.00	21	17.0
3010116	1.00	20.00	55	33.00	6.00	21	17.0

3020223	2.00	11.00	53	52.00	5.00	21	17.0
2020208	2.00	20.00	46	10.00	4.00	18	17.0
2010147	1.00	15.00	50	11.00	3.00	23	18.0
2010246	2.00	17.00	56	11.00	2.00	20	19.0
2020223	2.00	20.00	60	33.00	4.00	22	20.0
1020129	1.00	20.00	56	9.00	2.00	21	19.0
3020209	2.00	20.00	60	20.00	1.00	25	20.0
3010225	2.00	20.00	58	30.00	4.00	22	22.0
3010210	2.00	20.00	53	9.00	4.00	22	21.0
2010225	2.00	15.00	42	10.00	2.00	25	20.0
3010231	2.00	20.00	58	30.00	5.00	22	19.0
2010137	1.00	20.00	52	23.00	7.00	18	19.0
2020134	1.00	20.00	52	24.00	3.00	22	19.0
3010229	2.00	20.00	49	14.00	6.00	21	19.0
1010224	2.00	18.00	48	57.00	7.00	23	19.0
1020131	1.00	16.00	51	54.00	4.00	27	18.0
3010224	2.00	20.00	46	27.00	2.00	24	18.0
1020117	1.00	20.00	45	27.00	3.00	15	18.0
2020210	2.00	20.00	58	8.00	5.00	24	17.0
3010127	1.00	20.00	52	10.00	8.00	16	17.0

2020139	1.00	17.00	52	38.00	4.00	26	18.0
3020232	2.00	20.00	58	18.00	8.00	29	18.0
3010133	1.00	20.00	55	31.00	2.00	20	17.0
1010101	1.00	20.00	0	0.00	9.00	18	20.0
2010135	1.00	20.00	52	17.00	4.00	17	19.0
3010234	2.00	20.00	50	48.00	6.00	18	19.0
2020218	2.00	17.00	49	40.00	4.00	19	19.0
3010107	1.00	16.00	44	9.00	4.00	17	19.0
1020225	2.00	20.00	53	26.00	5.00	23	18.0
3010239	2.00	20.00	51	43.00	2.00	22	17.0
3010101	1.00	20.00	54	33.00	8.00	23	15.0
3010216	2.00	20.00	51	53.00	6.00	11	15.0
3010125	1.00	20.00	44	45.00	4.00	19	15.0
3020208	2.00	20.00	60	27.00	8.00	25	25.0
3020203	2.00	15.00	60	54.00	4.00	16	21.0
2020231	2.00	18.00	52	20.00	4.00	20	21.0
2010222	2.00	20.00	55	39.00	3.00	28	20.0
2010231	2.00	20.00	55	37.00	3.00	28	20.0
1020127	1.00	20.00	53	28.00	5.00	22	20.0
2020212	2.00	20.00	48	27.00	7.00	22	20.0

2010235	2.00	20.00	42	50.00	3.00	20	20.0
1010227	2.00	20.00	56	8.00	3.00	26	19.0
2010239	2.00	20.00	55	24.00	3.00	20	19.0
1020219	2.00	20.00	51	57.00	4.00	15	19.0
1010228	2.00	20.00	51	34.00	9.00	18	19.0
2020124	1.00	18.00	49	25.00	4.00	23	19.0
2010138	1.00	20.00	48	44.00	3.00	13	19.0
1010111	1.00	20.00	45	0.00	4.00	25	19.0
3010103	1.00	12.00	54	49.00	4.00	20	18.0
3020236	2.00	16.00	47	49.00	4.00	12	18.0
1010216	2.00	20.00	44	10.00	8.00	20	18.0
1010225	2.00	20.00	38	50.00	3.00	10	18.0
3020204	2.00	19.00	60	35.00	4.00	23	17.0
2020102	1.00	20.00	51	53.00	7.00	21	17.0
3020104	1.00	20.00	51	12.00	8.00	22	17.0
2020122	1.00	14.00	45	52.00	3.00	22	17.0
2020129	1.00	20.00	45	56.00	3.00	19	17.0
2010224	2.00	20.00	48	48.00	9.00	25	16.0
2010141	1.00	20.00	51	32.00	5.00	23	19.0
1010145	1.00	14.00	49	45.00	3.00	17	19.0

3020239	2.00	20.00	53	28.00	5.00	26	19.0
3020107	1.00	20.00	51	53.00	5.00	15	19.0
3010227	2.00	20.00	55	50.00	6.00	12	18.0
1020122	1.00	20.00	57	13.00	6.00	18	17.0
1020113	1.00	20.00	55	36.00	9.00	24	17.0
3020136	1.00	20.00	51	43.00	3.00	22	17.0
3010104	1.00	20.00	43	50.00	1.00	24	17.0
2020204	2.00	20.00	57	35.00	3.00	11	16.0
2020230	2.00	15.00	47	44.00	4.00	17	16.0
2020206	2.00	20.00	55	17.00	6.00	18	18.0
3020109	1.00	20.00	58	50.00	6.00	24	19.0
3020102	1.00	20.00	52	56.00	5.00	24	19.0
1020229	2.00	20.00	52	34.00	4.00	17	21.0
1010244	2.00	20.00	58	45.00	6.00	33	20.0
3020115	1.00	20.00	58	0.00	0.00	24	20.0
1010230	2.00	20.00	49	6.00	0.00	17	18.0
3010230	2.00	14.00	48	58.00	4.00	20	20.0
1010140	1.00	20.00	53	59.00	3.00	13	19.0
2020126	1.00	20.00	55	45.00	6.00	22	18.0
1020220	2.00	16.00	46	18.00	3.00	13	18.0

3010228	2.00	14.00	55	21.00	3.00	17	17.0
2010133	1.00	15.00	52	21.00	2.00	19	17.0
3010114	1.00	20.00	60	18.00	6.00	18	16.0
1020207	2.00	20.00	50	1.00	9.00	24	16.0
1010236	2.00	20.00	48	32.00	3.00	18	16.0
2010150	1.00	17.00	52	22.00	2.00	22	15.0
2010212	2.00	20.00	51	30.00	6.00	29	15.0
3010134	1.00	15.00	46	38.00	2.00	18	19.0
3020205	2.00	20.00	42	47.00	4.00	21	20.0
3020229	2.00	20.00	54	0.00	3.00	23	20.0
2010123	1.00	20.00	50	40.00	3.00	25	20.0
3020227	2.00	20.00	57	50.00	4.00	24	222.0
3020101	1.00	20.00	55	55.00	5.00	24	21.0
2020135	1.00	17.00	46	18.00	3.00	16	21.0
1010229	2.00	20.00	59	13.00	5.00	24	20.0
1010126	1.00	20.00	41	45.00	5.00	17	20.0
3020103	1.00	20.00	53	28.00	3.00	24	18.0
3010118	1.00	20.00	48	1.00	9.00	16	18.0
2010125	1.00	20.00	47	41.00	4.00	24	18.0
3010119	1.00	20.00	45	59.00	5.00	16	18.0

2020107	1.00	20.00	37	46.00	9.00	15	18.0
1020110	1.00	20.00	49	8.00	7.00	27	17.0
3020132	1.00	20.00	47	13.00	6.00	24	17.0
3010106	1.00	20.00	40	29.00	5.00	17	17.0
3010205	2.00	20.00	32	4.00	5.00	12	16.0
3010110	1.00	20.00	59	0.00	7.00	25	15.0
2020220	2.00	20.00	55	38.00	4.00	28	13.0
1020228	2.00	20.00	52	14.00	2.00	22	23.0
1020120	1.00	20.00	46	0.00	5.00	22	23.0
1010137	1.00	16.00	59	32.00	4.00	23	22.0
1020218	2.00	16.00	47	44.00	4.00	18	22.0
1010135	1.00	20.00	48	0.00	5.00	22	21.0
1020126	1.00	15.00	58	52.00	5.00	19	20.0
3020123	1.00	20.00	16	37.00	5.00	21	20.0
3020134	1.00	28.00	55	47.00	5.00	24	19.0
2020217	2.00	20.00	53	27.00	4.00	22	19.0
1020133	1.00	11.00	53	69.00	2.00	19	19.0
1020132	1.00	20.00	50	40.00	5.00	20	18.0
3010131	1.00	20.00	53	15.00	8.00	17	20.0
2010104	1.00	20.00	53	20.00	6.00	25	20.0

1020101	1.00	20.00	50	31.00	5.00	22	20.0
2010132	1.00	20.00	50	40.00	6.00	17	19.0
2010149	1.00	20.00	49	23.00	4.00	19	19.0
2010107	1.00	17.00	46	30.00	4.00	23	17.0
1010129	1.00	20.00	34	13.00	4.00	24	21.0
1010124	1.00	20.00	54	23.00	5.00	23	20.0
1010202	2.00	20.00	52	22.00	3.00	21	20.0
1020210	2.00	20.00	52	9.00	5.00	19	20.0
1020217	2.00	15.00	52	13.00	4.00	21	20.0
3020220	2.00	20.00	50	56.00	3.00	24	20.0
2010232	2.00	15.00	54	53.00	4.00	24	19.0
3010211	2.00	20.00	54	29.00	8.00	7	19.0
2010124	1.00	20.00	49	50.00	6.00	14	19.0
3010126	1.00	20.00	16	28.00	4.00	22	19.0
1010240	2.00	20.00	55	30.00	6.00	16	18.0
3010105	1.00	20.00	51	14.00	10.00	16	18.0
2010217	2.00	20.00	50	12.00	4.00	23	18.0
2020205	2.00	20.00	0	40.00	4.00	20	18.0
2020222	2.00	19.00	48	35.00	2.00	24	17.0
3020137	1.00	18.00	46	46.00	3.00	22	17.0

1010118	1.00	20.00	48	56.00	4.00	23	16.0
3010222	2.00	20.00	54	54.00	7.00	15	15.0
2010240	2.00	17.00	49	56.00	3.00	20	16.0
1020223	2.00	20.00	48	0.00	3.00	20	18.0
1020231	2.00	20.00	53	34.00	4.00	14	19.0
2010101	1.00	20.00	53	27.00	3.00	14	21.0
3010130	1.00	20.00	46	24.00	3.00	6	21.0
1010139	1.00	20.00	57	50.00	4.00	17	20.0
2010238	2.00	20.00	55	40.00	7.00	22	20.0
2010236	2.00	20.00	55	10.00	3.00	31	20.0
1010205	2.00	20.00	52	0.00	0.00	21	20.0
3020222	2.00	20.00	51	22.00	3.00	22	20.0
2020118	1.00	20.00	58	41.00	6.00	24	19.0
1020109	1.00	20.00	51	18.00	7.00	18	19.0
2020117	1.00	20.00	44	22.00	7.00	22	18.0
2010230	2.00	19.00	52	32.00	3.00	30	17.0
3010212	2.00	20.00	48	52.00	4.00	17	17.0
3020234	2.00	20.00	34	53.00	5.00	20	18.0
2020128	1.00	20.00	52	23.00	6.00	19	20.0
3010236	2.00	20.00	45	8.00	4.00	20	20.0

1010203	2.00	20.00	57	0.00	4.00	18	21.0
2010117	1.00	20.00	40	28.00	7.00	23	21.0
2010126	1.00	20.00	50	0.00	3.00	15	20.0
2010227	2.00	20.00	54	32.00	6.00	22	19.0
2010140	1.00	20.00	49	26.00	3.00	18	19.0
1020214	2.00	17.00	44	38.00	3.00	18	19.0
1020121	1.00	20.00	57	48.00	6.00	15	18.0
1010134	1.00	20.00	50	2.00	5.00	11	17.0
2020133	1.00	20.00	50	9.00	3.00	22	17.0
1020221	2.00	15.00	46	28.00	5.00	12	17.0
3020217	2.00	20.00	46	48.00	4.00	12	16.0
2020234	2.00	20.00	56	40.00	5.00	21	15.0
2010142	2.00	20.00	49	42.00	6.00	18	16.0
1010142	1.00	20.00	48	37.00	3.00	22	17.0
3020130	1.00	20.00	49	0.00	8.00	23	20.0
2010219	2.00	20.00	53	15.00	5.00	27	20.0
3020212	2.00	20.00	50	38.00	4.00	16	22.0
1010237	2.00	20.00	30	0.00	5.00	22	22.0
2020226	2.00	20.00	56	10.00	8.00	23	20.0
2010234	2.00	19.00	45	10.00	4.00	31	18.0

2020213	2.00	20.00	51	9.00	14.00	10	17.0
2010148	1.00	18.00	46	39.00	0.00	8	17.0
2020109	1.00	16.00	49	12.00	3.00	13	16.0
1010239	2.00	20.00	36	52.00	9.00	20	16.0
3010113	1.00	20.00	19	28.00	10.00	12	16.0
3010233	2.00	20.00	48	54.00	3.00	20	21.0
1010128	1.00	20.00	55	37.00	5.00	22	20.0
2020132	1.00	17.00	50	15.00	3.00	21	19.0
3010213	2.00	20.00	48	25.00	6.00	9	18.0
2020111	1.00	14.00	44	0.00	5.00	23	14.0
2020106	1.00	20.00	54	58.00	4.00	13	11.0
3020111	1.00	20.00	47	4.00	6.00	21	17.0
3020118	1.00	20.00	47	50.00	6.00	21	19.0
3020213	2.00	20.00	38	31.00	5.00	19	19.0
1010201	2.00	20.00	50	0.00	6.00	18	17.0
3020108	1.00	20.00	46	52.00	6.00	17	17.0
3020114	1.00	20.00	46	38.00	9.00	17	16.0
3010242	2.00	20.00	57	8.00	3.00	27	12.0
2010233	2.00	20.00	55	56.00	5.00	20	21.0
2020224	2.00	20.00	59	11.00	5.00	14	19.0

3020207	2.00	20.00	50	25.00	4.00	22	18.0
2020131	1.00	17.00	48	21.00	3.00	16	18.0
3010120	1.00	20.00	36	14.00	7.00	15	18.0
1010113	1.00	20.00	52	27.00	10.00	24	17.0
3020218	2.00	20.00	58	43.00	3.00	23	16.0
3010206	1.00	20.00	43	43.00	4.00	22	15.0
3010202	2.00	20.00	43	18.00	7.00	22	15.0
3020210	2.00	20.00	51	1.00	6.00	18	16.0
3020215	2.00	20.00	0	48.00	11.00	14	22.0
2010218	2.00	20.00	49	25.00	5.00	23	21.0
1010222	2.00	20.00	48	49.00	3.00	29	20.0
2020137	1.00	20.00	48	34.00	4.00	15	20.0
3020238	2.00	20.00	44	56.00	4.00	25	20.0
3020219	2.00	20.00	46	43.00	4.00	22	19.0
1010115	1.00	20.00	45	26.00	0.00	22	19.0
1010108	1.00	20.00	56	54.00	0.00	15	18.0
2020116	1.00	20.00	53	34.00	5.00	18	18.0
1010214	2.00	33.00	51	36.00	6.00	20	17.0
3010111	1.00	20.00	39	14.00	6.00	17	16.0
1020208	2.00	20.00	59	20.00	5.00	19	22.0

1010110	1.00	20.00	28	12.00	6.00	26	21.0
2010229	2.00	20.00	51	40.00	6.00	28	18.0
2010130	1.00	16.00	48	45.00	8.00	12	18.0
1010217	2.00	20.00	47	3.00	4.00	13	18.0
2020207	2.00	20.00	40	49.00	13.00	21	18.0
3020237	2.00	20.00	54	40.00	7.00	26	17.0
3010117	1.00	20.00	54	46.00	5.00	15	20.0
1010223	2.00	20.00	49	40.00	4.00	23	20.0
1010207	2.00	20.00	46	0.00	0.00	14	17.0
3010102	1.00	20.00	35	20.00	5.00	22	16.0
1010242	2.00	17.00	56	40.00	4.00	26	13.0
3020221	2.00	17.00	49	17.00	4.00	19	21.0
2010223	2.00	20.00	55	10.00	6.00	17	20.0
1020114	1.00	20.00	17	23.00	15.00	12	18.0
2020101	1.00	20.00	49	20.00	4.00	17	17.0
3010122	1.00	20.00	52	36.00	6.00	15	13.0
2020119	1.00	20.00	36	38.00	9.00	21	20.0
1010235	2.00	20.00	49	15.00	5.00	15	19.0
3020125	1.00	20.00	49	20.00	10.00	12	19.0
1010106	1.00	20.00	47	13.00	7.00	15	18.0

2010228	2.00	20.00	42	30.00	9.00	19	17.0
1010117	1.00	20.00	37	15.00	3.00	18	16.0
2010207	2.00	20.00	43	20.00	15.00	20	15.0
2020136	1.00	20.00	46	48.00	9.00	21	20.0
2010127	1.00	20.00	40	18.00	7.00	15	20.0
1010206	2.00	20.00	38	0.00	0.00	11	19.0
1020134	1.00	20.00	58	20.00	3.00	19	18.0
1010109	1.00	20.00	46	24.00	4.00	21	18.0
3020120	1.00	20.00	35	13.00	0.00	17	18.0
3020113	1.00	20.00	35	40.00	6.00	17	18.0
3010201	2.00	20.00	13	20.00	7.00	8	18.0
1010103	1.00	20.00	19	49.00	6.00	12	17.0
1010144	1.00	20.00	47	47.00	5.00	23	13.0
2010116	1.00	20.00	33	45.00	9.00	15	11.0
3020230	2.00	20.00	54	0.00	5.00	24	21.0
2020120	1.00	20.00	53	26.00	8.00	22	21.0
2010220	2.00	20.00	50	52.00	7.00	25	21.0
1010132	1.00	15.00	48	56.00	4.00	21	19.0
1010130	1.00	20.00	54	30.00	7.00	16	18.0
2010216	2.00	20.00	42	21.00	6.00	26	17.0

2020112	1.00	20.00	0	8.00	8.00	7	17.0
2010205	2.00	20.00	54	45.00	8.00	19	16.0
2020108	1.00	3.00	0	0.00	5.00	7	16.0
2020203	2.00	18.00	41	46.00	5.00	12	14.0
3010109	1.00	17.00	44	34.00	2.00	20	15.0
2020229	2.00	20.00	50	47.00	3.00	18	16.0
2020110	1.00	20.00	39	2.00	9.00	18	18.0
1010136	1.00	19.00	46	9.00	3.00	20	16.0
1010114	1.00	17.00	51	46.00	3.00	9	15.0
3010124	1.00	20.00	32	15.00	3.00	8	19.0
1020205	2.00	20.00	30	48.00	5.00	12	19.0
1010123	1.00	20.00	32	33.00	6.00	16	17.0
3010112	1.00	2.00	0	30.00	4.00	7	15.0
2020125	1.00	20.00	54	0.00	4.00	17	20.0
1010112	1.00	20.00	39	18.00	5.00	20	20.0
3010203	2.00	20.00	36	28.00	8.00	14	20.0
2010226	2.00	20.00	35	57.00	4.00	17	19.0
3020128	1.00	15.00	50	55.00	4.00	18	18.0
3010123	1.00	20.00	37	53.00	6.00	10	18.0
2010105	1.00	20.00	35	10.00	6.00	10	18.0

2020121	1.00	13.00	54	22.00	4.00	17	17.0
1010215	2.00	15.00	52	35.00	5.00	15	17.0
1020203	2.00	20.00	54	27.00	8.00	17	16.0
2010111	1.00	20.00	21	0.00	9.00	21	16.0
1010221	2.00	15.00	40	39.00	5.00	20	19.0
3010204	2.00	20.00	30	53.00	4.00	10	19.0
1010127	1.00	20.00	49	46.00	13.00	22	17.0
2010114	1.00	20.00	29	5.00	5.00	10	17.0
1010234	2.00	15.00	57	5.00	3.00	21	16.0
1020103	1.00	20.00	29	3.00	7.00	14	19.0
2010119	1.00	20.00	36	9.00	7.00	15	18.0
2020130	1.00	20.00	42	11.00	8.00	20	23.0
1010238	2.00	20.00	33	1.00	5.00	15	21.0
2010213	2.00	20.00	56	7.00	7.00	25	19.0
1020115	1.00	20.00	42	0.00	7.00	18	19.0
1010125	1.00	20.00	33	49.00	11.00	15	18.0
1010220	2.00	20.00	40	24.00	4.00	10	16.0
3020202	2.00	20.00	60	38.00	5.00	20	19.0
3020233	0.00	20.00	49	15.00	4.00	12	20.0
3020112	1.00	15.00	48	19.00	4.00	14	18.0

1010219	2.00	20.00	21	17.00	6.00	15	17.0
1010121	1.00	20.00	23	45.00	0.00	7	16.0
1020206	2.00	20.00	42	20.00	5.00	10	15.0
3020119	1.00	17.00	48	8.00	6.00	14	14.0
1020108	1.00	20.00	34	2.00	9.00	9	14.0
1020213	2.00	20.00	33	30.00	7.00	9	14.0
2010204	2.00	20.00	59	23.00	7.00	26	16.0
3010215	2.00	20.00	28	30.00	7.00	10	15.0
2010201	2.00	20.00	50	40.00	7.00	20	15.0
3020117	1.00	20.00	50	20.00	5.00	10	16.0
2010109	1.00	16.00	46	38.00	5.00	15	13.0
2020104	1.00	20.00	35	30.00	7.00	6	13.0
3020110	1.00	20.00	50	16.00	6.00	10	17.0
1020204	2.00	20.00	49	14.00	13.00	10	16.0
1010119	1.00	20.00	42	5.00	5.00	14	16.0
1010116	1.00	20.00	49	0.00	4.00	17	18.0
2010103	1.00	20.00	24	20.00	3.00	11	16.0
1010131	1.00	20.00	32	9.00	6.00	20	19.0
1010231	2.00	20.00	14	15.00	6.00	7	16.0
1020212	2.00	20.00	45	20.00	7.00	15	22.0

2010211	2.00	20.00	34	25.00	3.00	11	15.0
2010214	2.00	20.00	47	18.00	7.00	17	18.0
1020209	2.00	14.00	27	9.00	5.00	17	18.0
1020112	1.00	20.00	43	14.00	0.00	0	17.0
2010208	2.00	20.00	39	32.00	9.00	19	15.0
1010141	1.00	20.00	38	50.00	5.00	7	16.0
1010226	2.00	20.00	55	13.00	4.00	13	15.0
2020103	1.00	20.00	17	1.00	14.00	8	15.0
2010120	1.00	20.00	56	56.00	5.00	18	18.0
2010112	1.00	20.00	0	0.00	0.00	8	20.0
3020216	2.00	20.00	48	30.00	6.00	18	16.0
2020209	2.00	15.00	52	2.00	3.00	15	21.0
1010210	2.00	20.00	0	40.00	5.00	6	17.0
2010210	2.00	20.00	18	58.00	13.00	23	10.0
3010221	2.00	20.00	30	20.00	6.00	7	20.0
1010218	2.00	20.00	30	43.00	4.00	10	16.0
2020115	1.00	20.00	26	12.00	5.00	6	15.0
1010233	2.00	20.00	21	16.00	4.00	13	16.0
2010102	1.00	20.00	37	49.00	5.00	8	14.0
1020107	1.00	20.00	42	1.00	5.00	9	14.0

1010122	1.00	20.00	0	15.00	0.00	0	14.0
3010121	1.00	2.00	0	13.00	2.00	0	17.0
3010207	2.00	20.00	21	6.00	4.00	4	15.0
1020105	1.00	20.00	37	17.00	7.00	8	14.0
2010110	1.00	20.00	8	5.00	7.00	4	17.0
1010209	2.00	0.00	0	0.00	0.00	5	16.0
2020202	2.00	12.00	0	50.00	5.00	5	16.0
1010133	1.00	20.00	11	11.00	7.00	6	19.0
1010211	2.00	20.00	0	10.00	6.00	4	15.0
3020124	1.00	20.00	0	46.00	4.00	7	20.0
2020114	1.00	20.00	35	0.00	7.00	2	13.0
1020201	2.00	20.00	40	15.00	6.00	9	19.0
2020214	2.00	20.00	38	50.00	8.00	6	16.0
2020113	1.00	0.00	0	35.00	6.00	6	17.0
2020201	2.00	20.00	0	32.00	10.00	0	17.0
2010206	2.00	20.00	17	2.00	11.00	0	19.0
2010215	2.00	20.00	40	20.00	2.00	7	18.0
2010113	1.00	20.00	0	0.00	0.00	4	16.0
1010105	1.00	20.00	38	29.00	6.00	8	13.0
2010202	2.00	20.00	45	50.00	6.00	12	17.0

3020121	1.00	12.00	0	52.00	5.00	13	14.0
2020219	2.00	20.00	20	31.00	7.00	4	21.0
1020106	1.00	20.00	33	52.00	5.00	4	16.0
1010212	2.00	20.00	0	0.00	2.00	5	10.0
1020111	1.00	20.00	31	21.00	5.00	5	19.0
1010102	1.00	20.00	6	11.00	7.00	4	13.0
1020116	1.00	20.00	44	1.00	6.00	6	8.0
1020202	2.00	20.00	38	0.00	6.00	6	15.0
2010131	1.00	20.00	35	1.00	7.00	5	18.0
1010120	1.00	20.00	41	0.00	8.00	5	16.0
3020126	1.00	19.00	0	34.00	3.00	0	8.0
1010104	1.00	20.00	0	0.00	0.00	0	6.0
1010208	2.00	20.00	44	0.00	0.00	0	15.0
2010118	1.00	20.00	0	51.00	0.00	7	10.0
3020122	1.00	20.00	0	49.00	8.00	8	10.0
2010115	1.00	20.00	0	25.00	0.00	1	6.0
2010108	1.00	17.00	52	14.00	0.00	0	18.0
2010106	1.00	20.00	52	14.00	3.00	0	17.0
2010203	2.00	20.00	0	0.00	0.00	9	7.0
1020104	1.00	20.00	7	2.00	4.00	0	11.0

1020102	1.00	20.00	15	55.00	6.00	2	18.0
2010209	2.00	20.00	0	0.00	0.00	0	9.0
3010214	2.00	20.00	0	0.00	0.00	0	9.0
2020105	1.00	0.00	0	0.00	0.00	0	8.0
1010138	1.00	20.00	48	29.00	7.00	0	15.0
