# Supplementary material for: Reliability and validity of Handwriting Test for Preschool Children (HT-PRE): A new tool to assess the handwriting ability of preschool children aged 5–6 years old in Mainland China
Source: PLoS One. 2020 Mar 2;15(3):e0229786. doi: 10.1371/journal.pone.0229786 (PMC7051084; doi:10.1371/journal.pone.0229786)
Supplement: S3 Table — 49 cases of children with handwriting difficulties, as recognized by the clinical observation of attending physicians, were randomly selected from the children's psychological behavior clinics at Nanjing Maternal and Child Health Care Hospital in order to participate in this study. (PDF) [file pone.0229786.s003.pdf]

ratio score	number score	letter score	letter time	characters score	characters time
6.0	45.0	22.0	196.0	4.0	273.0
2.0	28.0	20.0	211.0	0.0	320.0
0.0	0.0	4.0	144.0	4.0	177.0
4.0	15.0	14.0	178.0	0.0	267.0
0.0	0.0	11.0	415.0	3.0	351.0
5.0	39.0	19.0	160.0	3.0	203.0
5.0	41.0	34.0	111.0	2.0	397.0
3.0	39.0	21.0	414.0	4.0	153.0
6.0	39.0	9.0	342.0	1.0	376.0
3.0	30.0	18.0	364.0	0.0	282.0
6.0	44.0	19.0	110.0	4.0	261.0
5.0	27.0	40.0	165.0	9.0	540.0
5.0	36.0	32.0	342.0	6.0	489.0
5.0	49.0	42.0	364.0	11.0	638.0
4.0	36.0	0.0	110.0	0.0	100.0
9.0	53.0	43.0	165.0	20.0	267.0
9.0	43.0	54.0	464.0	27.0	304.0
8.0	53.0	18.0	108.0	2.0	178.0
9.0	38.0	41.0	144.0	3.0	465.0
4.0	43.0	42.0	407.0	12.0	467.0
4.0	53.0	65.0	135.0	21.0	230.0
7.0	41.0	46.0	120.0	11.0	338.0
9.0	46.0	52.0	122.0	0.0	220.0
5.0	33.0	42.0	287.0	9.0	198.0
9.0	38.0	58.0	143.0	15.0	422.0
4.0	41.0	17.0	192.0	5.0	323.0
9.0	35.0	54.0	95.0	8.0	265.0
8.0	45.0	34.0	232.0	7.0	319.0
6.0	42.0	56.0	230.0	10.0	350.0
7.0	46.0	41.0	190.0	19.0	370.0
9.0	49.0	65.0	137.0	26.0	230.0
5.0	40.0	48.0	137.0	9.0	318.0
8.0	49.0	56.0	89.0	13.0	369.0

6.0	52.0	47.0	100.0	6.0	165.0
7.0	40.0	50.0	267.0	13.0	342.0
8.0	37.0	45.0	304.0	15.0	364.0
5.0	35.0	32.0	178.0	7.0	210.0
6.0	36.0	45.0	368.0	18.0	165.0
8.0	23.0	35.0	370.0	14.0	464.0
9.0	54.0	49.0	76.0	22.0	297.0
8.0	53.0	23.0	342.0	17.0	220.0
6.0	46.0	44.0	364.0	19.0	110.0
7.0	49.0	41.0	110.0	21.0	165.0
7.0	40.0	36.0	165.0	17.0	200.0
8.0	49.0	39.0	200.0	14.0	332.0
9.0	52.0	59.0	132.0	22.0	319.0
8.0	37.0	52.0	198.0	10.0	210.0
9.0	41.0	42.0	234.0	20.0	387.0
9.0	42.0	49.0	189.0	15.0	399.0
